# Supplementary figures and images for: HCMV encoded UL84 hijacks FHL2 to suppress type I interferon production and enhance viral replication
Source: PLoS Pathog. 2026 Jan 26;22(1):e1013895. doi: 10.1371/journal.ppat.1013895 (PMC12863675; doi:10.1371/journal.ppat.1013895)

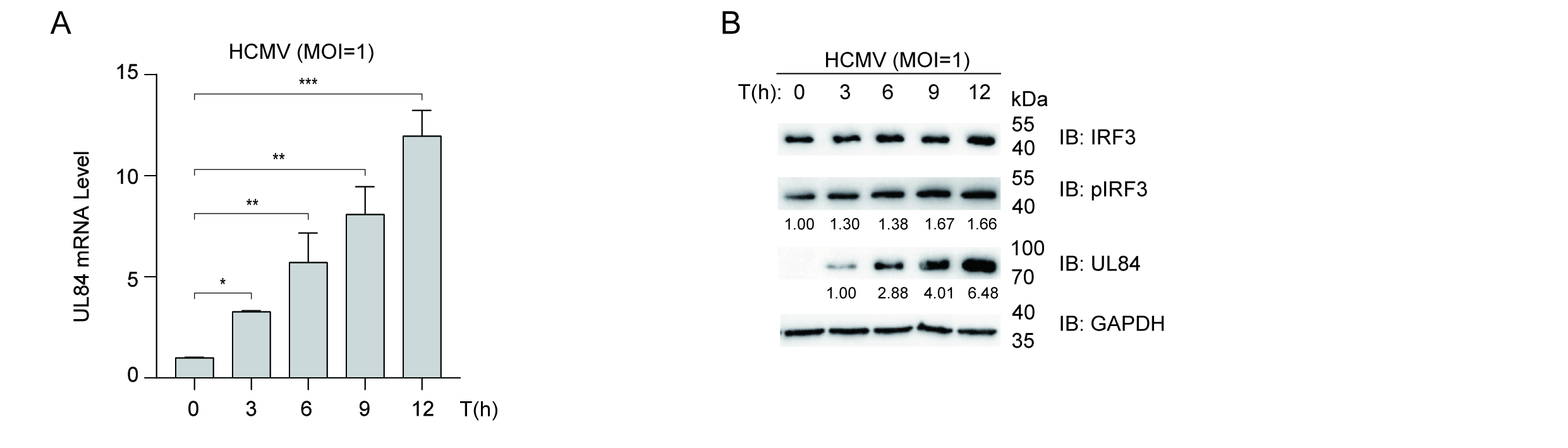

Supplement: S1 Fig — (A) HCMV (MOI = 1) for the indicated times before RT-qPCR analysis of the indicated UL84 genes in HFF cells. (B) HCMV (MOI = 1) for the indicated times before western blot analysis of the indicated UL84, IRF3 and pIRF3 in HFF cells. All experimental assays were conducted in triplicate with independent biological replicates. For all figures, statistical analyses were performed using two-tailed t-test. Differences were considered statistically significant when * denoted p < 0.05, ** denoted p < 0.01, *** denoted p < 0.001, and **** denoted p < 0.0001. (TIF) [file ppat.1013895.s001.tif]

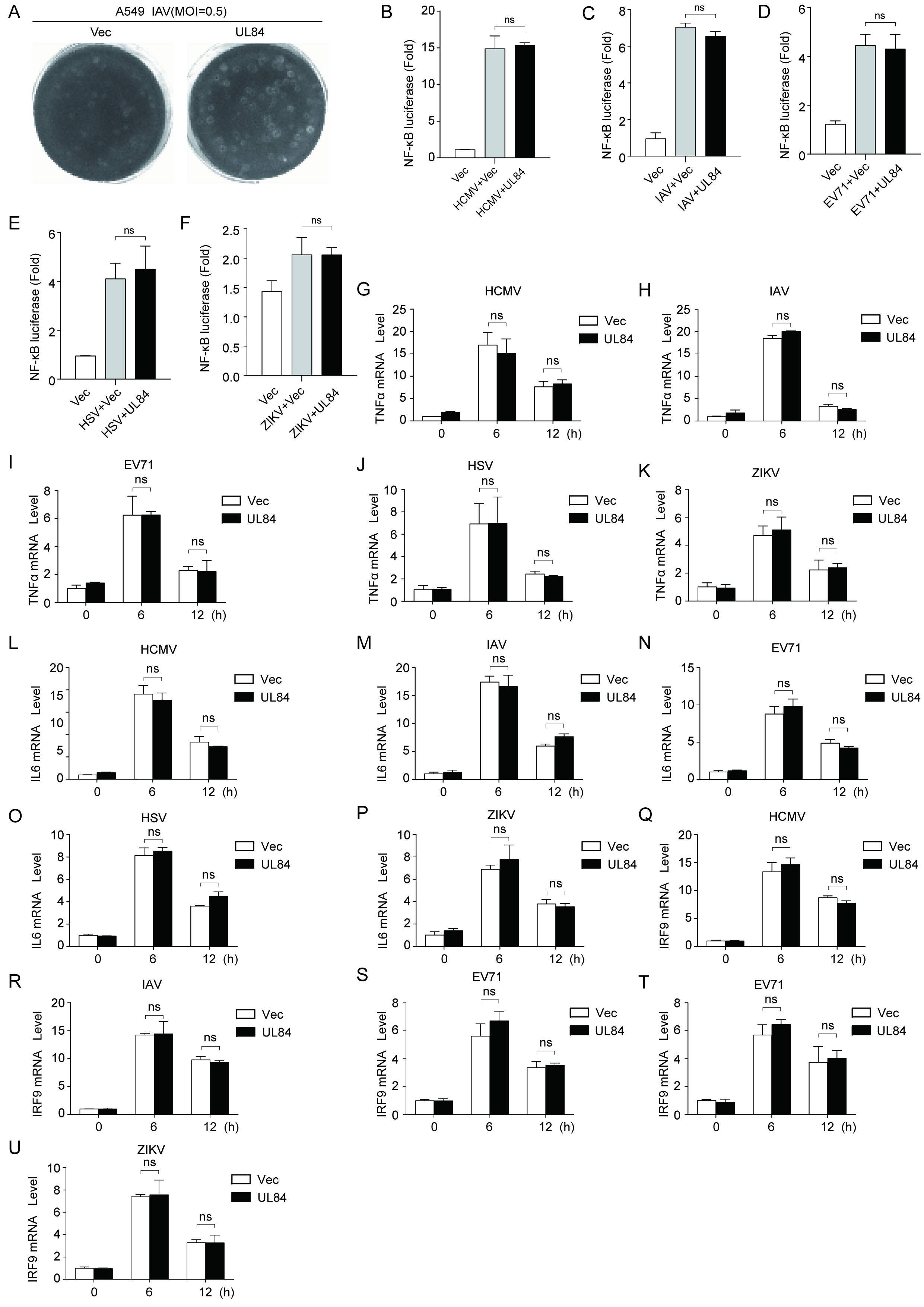

Supplement: S2 Fig — (A) A549 cells were first transfected with the UL84-expressing plasmid. After 24 hours, cells were infected with IAV (MOI = 0.5). All experimental assays were conducted in triplicate with independent biological replicates. (B) (C) (D) (E) (F) HFF cells were transfected with pGL3-Luci-NF-κB and pRL-TK plasmids, along with increasing amounts of pcDNA3.1-UL84 expression vectors for 24 h. Then, HFFs infected with HCMV (MOI = 1), IAV (MOI = 0.5), EV71 (MOI = 1), HSV (MOI = 1), or ZIKV (MOI = 1) for an additional 24 h, after which dual-luciferase reporter assays were conducted. All experimental assays were conducted in triplicate with independent biological replicates. (G) (H) (I) (J) (K) HFF cells were transfected with the plasmid pcDNA3.1-UL84 or the empty vector for 24 h, and then respectively infected with HCMV (MOI = 1), IAV (MOI = 0.5), EV71 (MOI = 1), HSV (MOI = 1), or ZIKV (MOI = 1) for the indicated times before RT-qPCR analysis of the indicated TNFα genes. All experimental assays were conducted in triplicate with independent biological replicates. (L) (M) (N) (O) (P) HFF cells were transfected with the plasmid pcDNA3.1-UL84 or the empty vector for 24 h, then respectively infected with HCMV (MOI = 1), IAV (MOI = 0.5), EV71 (MOI = 1), HSV (MOI = 1), or ZIKV (MOI = 1) for the indicated times before RT-qPCR analysis of the indicated IL6 genes. (Q) (R) (S) (T) (U) HFF cells were transfected with the plasmid pcDNA3.1-UL84 or the empty vector for 24 h, then respectively infected with HCMV (MOI = 1), IAV (MOI = 0.5), EV71 (MOI = 1), HSV (MOI = 1), or ZIKV (MOI = 1) for the indicated times before RT-qPCR analysis of the indicated IRF9 genes. For all figures, statistical analyses were performed using two-tailed t-test. Differences were considered statistically significant when * denoted p < 0.05, ** denoted p < 0.01, *** denoted p < 0.001, and **** denoted p < 0.0001. (TIF) [file ppat.1013895.s002.tif]

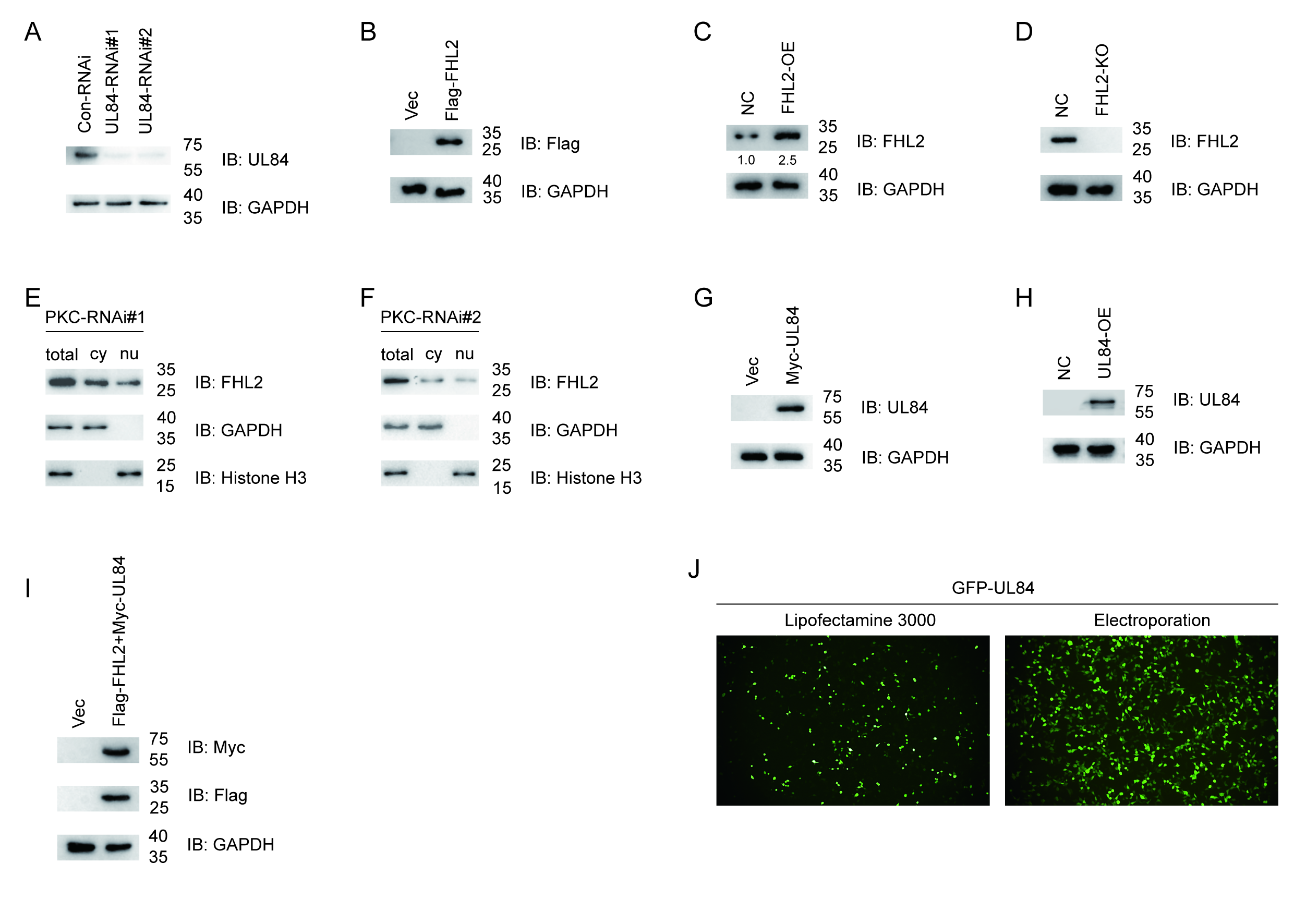

Supplement: S3 Fig — (A) UL84-RNAi-#1 or UL84-RNAi-#2 were transiently transfected in HFFs for 24 h, respectively. HFF cells were transiently transfected via electroporation, then infected with HCMV (MOI = 1) before Western blotting. (B) Expression plasmids of Flag-FHL2 were transiently transfected into HEK293T cells for 48 h, and then the total cell lysates were prepared and immunoprecipitated with anti-Flag antibody, followed by immunoblot analysis. (C) Immunoblotting analysis was performed using anti-FHL2 antibody on lysates from the FHL2-overexpressing cell line generated in HFF cells. (D) Immunoblotting analysis was performed using anti-FHL2 antibody on lysates from the FHL2-KO cell line generated in HFF cells. (E) (F) PKC-RNAi-#1 or PKC-RNAi-#2 were transiently transfected in HFFs for 24 h, respectively. HFF cells were transiently transfected via electroporation, then infected with HCMV (MOI = 1) before Western blotting. (G) Expression plasmids of Myc-UL84 were transiently transfected into HEK293T cells for 48 h, and then the total cell lysates were prepared and immunoprecipitated with anti-Myc antibody, followed by immunoblot analysis. (H) Immunoblotting analysis was performed using anti-UL84 antibody on lysates from the UL84-overexpressing cell line generated in HFF cells. The displayed images were representative ones from three independent experiments. (I) Expression plasmids of Flag-FHL2 and Myc-UL84 were transiently transfected into HEK293T cells for 48 h, and then the total cell lysates were prepared and immunoprecipitated with anti-Flag, anti-Myc antibodies, followed by immunoblot analysis. (J) HFF cells were transiently transfected vector via electroporation and Lipofectamine 3000 for 48 hours. Transfection results were observed under a fluorescence microscope 48 hours after transfection. The displayed images were representative ones from three independent experiments. (TIF) [file ppat.1013895.s003.tif]

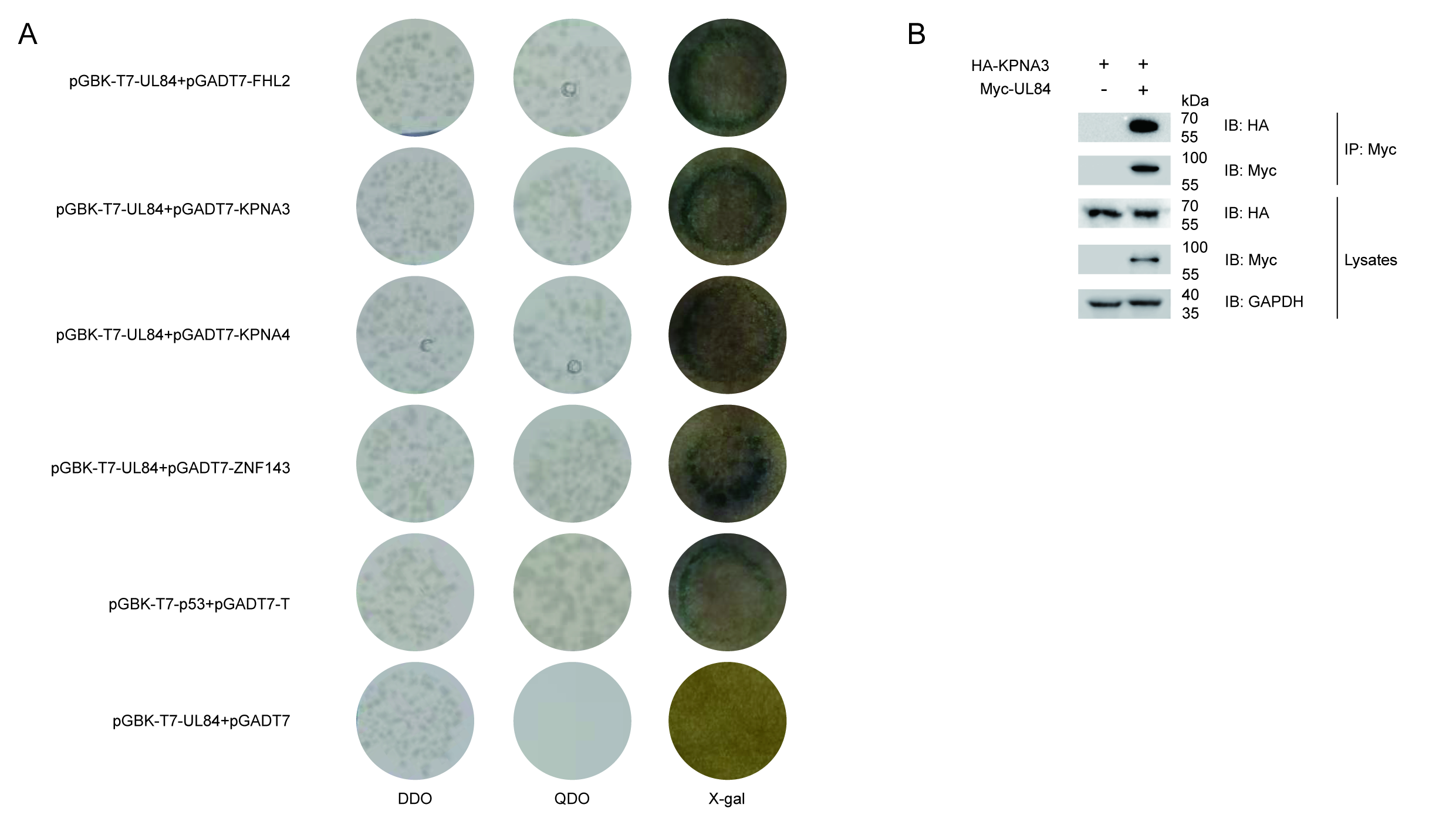

Supplement: S4 Fig — (A) The pGBK-T7-UL84 plasmid was first introduced into the AH109 strain to exclude the self-activation of reporter genes. The AH109 strain was then co-transformed with the pGBK-T7-UL84 plasmid with pGACT7-FHL2, pGACT7-KPNA3, pGACT7-KPNA4, and pGACT7-ZNF143. The AH109 strain was then co-transformed with the pGBKT7-UL84 plasmid. As a negative control, the AH109 strain was co-transformed with the pGBKT7 empty vector and the cDNA library plasmids. (B) Co-IP assays between UL84 and target proteins KPNA3. HEK293T cells were transiently plasmids as indicated for 48 h before Co-IP and immunoblots analysis. The displayed images were representative ones from three independent experiments. (TIF) [file ppat.1013895.s004.tif]

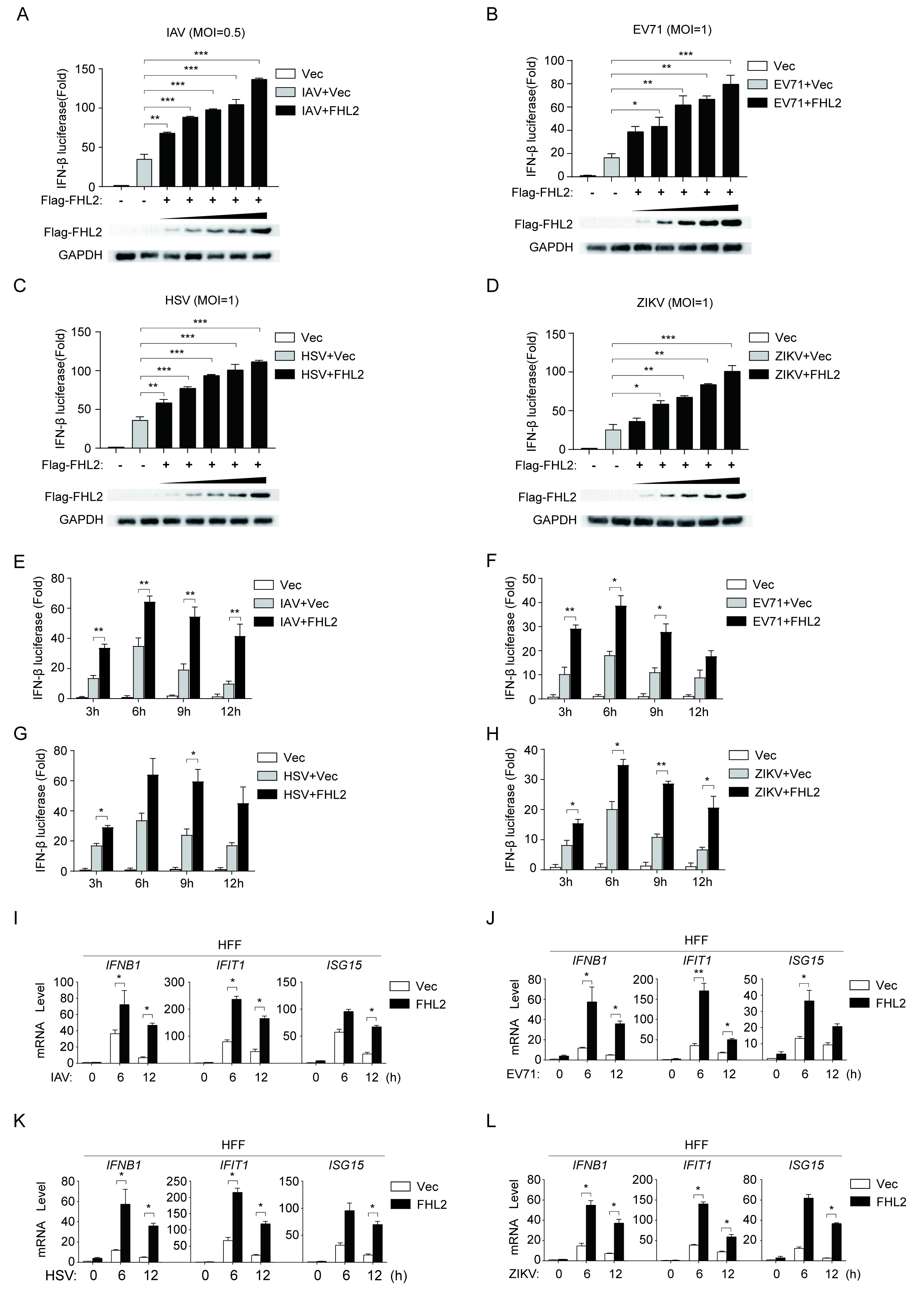

Supplement: S5 Fig — (A) (B) (C) (D) HFF cells were transfected with pGL3-luci-IFN-beta and pRL-TK plasmids, along with increasing amounts of pcDNA3.1-FHL2 expression vectors for 24 h. Then, HFFs infected with IAV (MOI = 0.5), EV71 (MOI = 1), HSV (MOI = 1), or ZIKV (MOI = 1) for an additional 6 h, after which dual-luciferase reporter assays were conducted. All experimental assays were conducted in triplicate with independent biological replicates. (E) (F) (G) (H) The pGL3-luci-IFN-beta and pcDNA3.1-FHL2 plasmids were transiently transfected into HFFs for 24 h. Dual-luciferase reporter assays were then conducted. All experimental assays were conducted in triplicate with independent biological replicates. (I) (J) (K) (L) FHL2 was transiently transfected into HFFs for 24 h. Then IAV (MOI = 0.5), EV71 (MOI = 1), HSV (MOI = 1), or ZIKV (MOI = 1) for different times as indicated. The expression of antiviral interferon-stimulated genes (IFIT1, ISG15 and IFNB1) was analyzed before RT-qPCR analysis, which was normalized to the mRNA level of GAPDH. All experimental assays were conducted in triplicate with independent biological replicates. For all figures, statistical analyses were performed using two-tailed t-test. Differences were considered statistically significant when * denoted p < 0.05, ** denoted p < 0.01, *** denoted p < 0.001, and **** denoted p < 0.0001. (TIF) [file ppat.1013895.s005.tif]

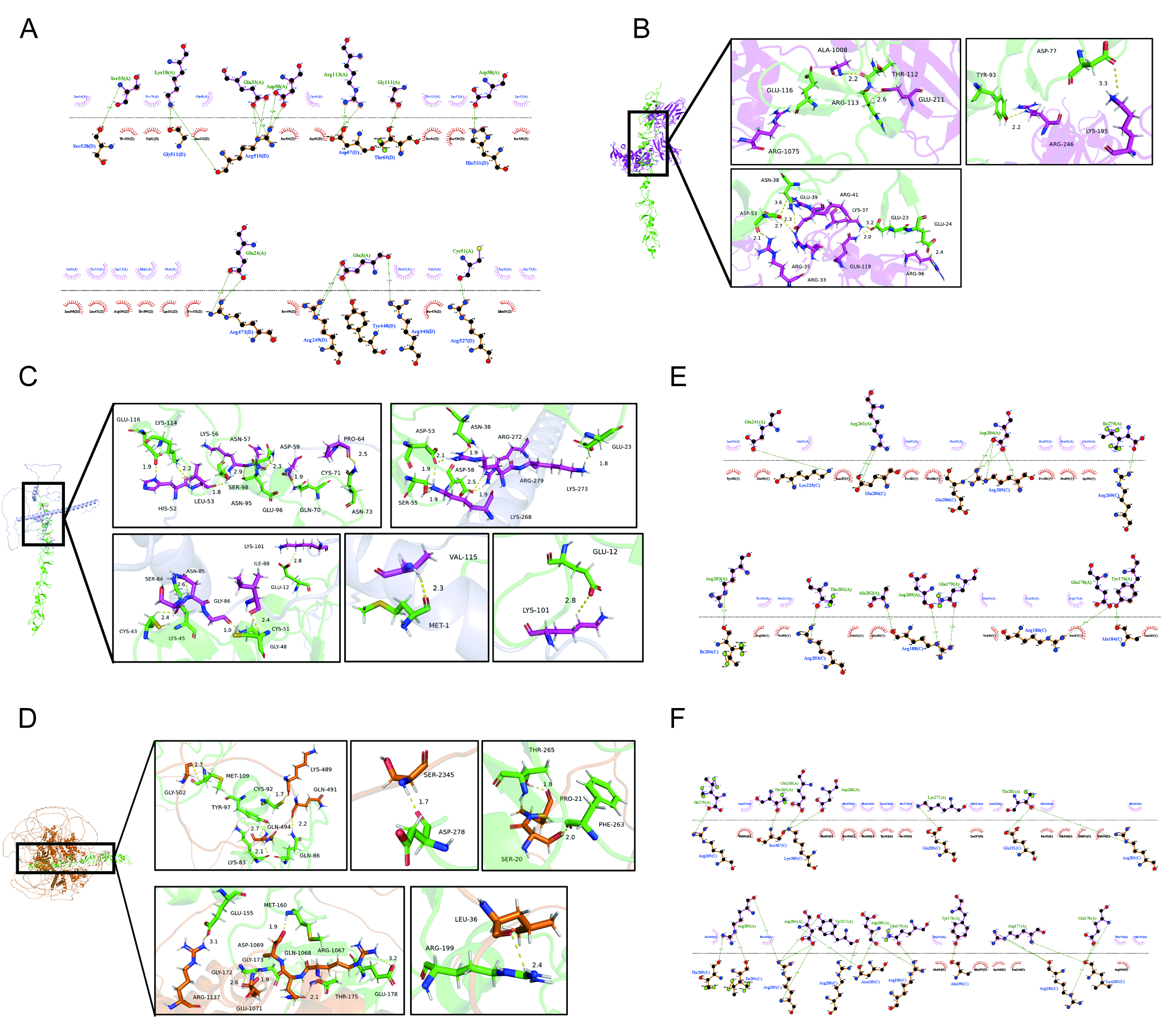

Supplement: S6 Fig — (A) Molecular docking of the UL84-FHL2 complex: 2D interaction diagram. (B) Molecular docking of the FHL2-IRF3 complex. Molecular docking simulation of protein FHL2 and IRF3. The structure of the IRF3, and FHL2 were downloaded from the Protein Data Bank, green refers to FHL2, purple refers to IRF3. (C) Molecular docking of the FHL2-c-Jun complex. The structure of the c-Jun, and FHL2 were downloaded from the Protein Data Bank, green refers to FHL2, wine red refers to c-Jun. (D) Molecular docking of the FHL2-p300 complex. The structure of the p300, and FHL2 were downloaded from the Protein Data Bank, green refers to FHL2, brown refers to p300. (E) Molecular docking of the IRF3-FHL2-TBP complex: 2D Interaction Diagram. (F) Molecular docking of the UL84-FHL2-TBP complex: 2D Interaction Diagram. (TIF) [file ppat.1013895.s006.tif]

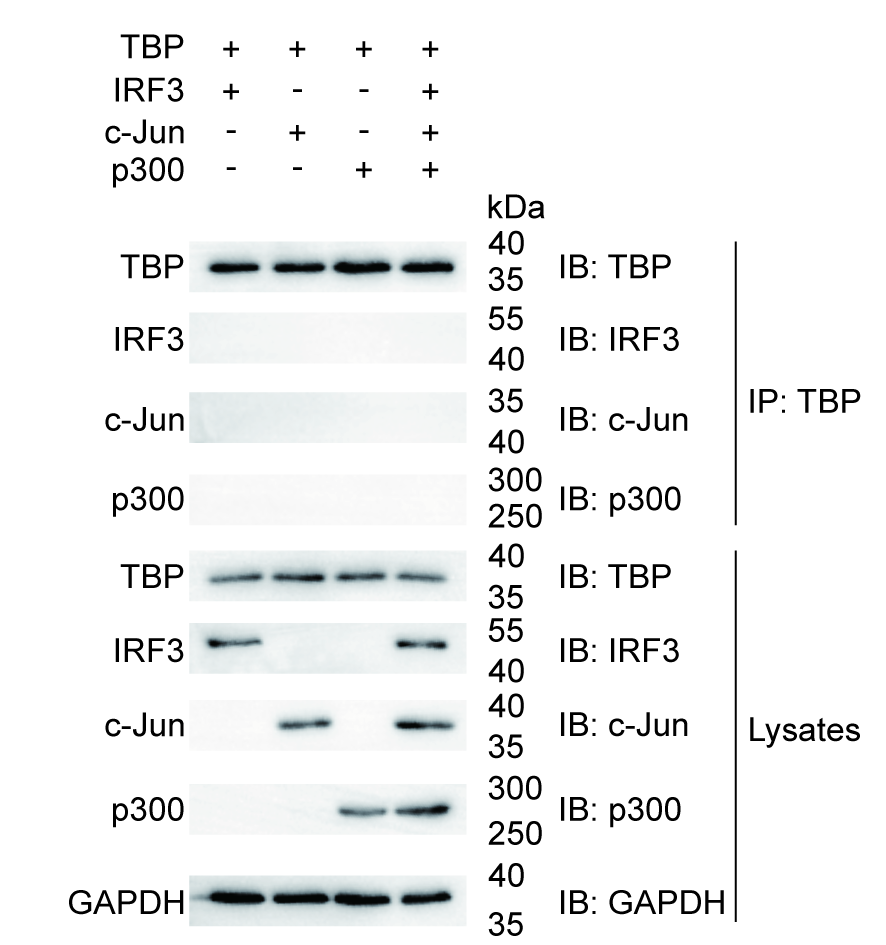

Supplement: S7 Fig — Expression plasmids of Flag-FHL2, TBP, IRF3, p300 and c-Jun were transiently transfected into HEK293T cells for 48 h, and then the total cell lysates were prepared and immunoprecipitated with anti-Flag, anti-TBP, anti-IRF3, anti-c-Jun or anti-p300 antibodies, followed by immunoblot analysis. The displayed images were representative ones from three independent experiments. (TIF) [file ppat.1013895.s007.tif]
