# Supplementary material for: HCMV encoded UL84 hijacks FHL2 to suppress type I interferon production and enhance viral replication
Source: PLoS Pathog. 2026 Jan 26;22(1):e1013895. doi: 10.1371/journal.ppat.1013895 (PMC12863675; doi:10.1371/journal.ppat.1013895)
Supplement: S1 Table — (DOCX) [file ppat.1013895.s008.docx]

**Supplementary Table 1. Yeast two-hybrid system screened interactions of viral proteins UL84 and Co-IP analysis.**

| **CMV Bait** | **Prey Gene Symbol** | **Prey**  **Gene ID** | **Prey Gene Name** | **Number**  **of hits** | **Primary Function** | **Secondary**  **Function** | **Notes** |
| --- | --- | --- | --- | --- | --- | --- | --- |
| **UL84** | FHL2 | 2274 | Four and a half LIM domains 2 | 6 | Regulation of transcription | Signal transduction | Confirmed by CO-IP |
|  | KPNA3 | 3839 | Karyopherin alpha 3 (importin alpha 3) | 5 | Nuclear protein import | Intracellular protein  transport | Confirmed by CO-IP |
|  | KPNA4 | 3840 | Karyopherin alpha 4 (importin alpha 4) | 1 | Nuclear protein import | Intracellular protein  transport |  |
|  | ZNF143 | 7702 | Zinc finger protein 143 | 1 | Regulation of transcription |  |  |

Yeast two-hybrid screening and Co-immunoprecipitation (Co-IP) validation of UL84-interacting host proteins. This table summarizes the host proteins identified by yeast two-hybrid (Y2H) screening using the HCMV protein UL84 as bait. The corresponding prey genes, gene IDs, number of positive clones (hits), and their annotated primary and secondary biological functions are listed. Proteins that showed reproducible interaction with UL84 were further validated by co-immunoprecipitation (Co-IP) assays.
