# Supplementary material for: HCMV encoded UL84 hijacks FHL2 to suppress type I interferon production and enhance viral replication
Source: PLoS Pathog. 2026 Jan 26;22(1):e1013895. doi: 10.1371/journal.ppat.1013895 (PMC12863675; doi:10.1371/journal.ppat.1013895)
Supplement: S2 Table — (DOCX) [file ppat.1013895.s009.docx]

**Supplementary Table 2. Plasmids used in this study.**

| Plasmids | Description | Reference |
| --- | --- | --- |
| pCMV-Myc | The empty vector with Myc tag |  |
| pRK5-Flag | The empty vector with Flag tag |  |
| pET28a-His | The empty vector with His tag |  |
| pGEX6p-1-GST | The empty vector with GST tag |  |
| pGL3-Luci-NF-κB | The empty vector with NF-κB promoter |  |
| pGL3-luci-IFN-beta | The empty vector with FN-β promoter |  |
| pCDNA3.1-UL84 | pCDNA3.1 containing HCMV UL84 full length sequence | This study |
| pCMV-Myc-UL84 | pCMV-Myc containing HCMV UL84 full length sequence | This study |
| pCMV-Myc-UL84(2-540) | pCMV-Myc containing HCMV UL84 truncated sequence (2-540aa) | This study |
| pCMV-Myc-UL84(2-460) | pCMV-Myc containing HCMV UL84 truncated sequence (2-460aa) | This study |
| pCMV-Myc-UL84(2-399) | pCMV-Myc containing HCMV UL84 truncated sequence (2-399aa) | This study |
| pCMV-Myc-UL84(400-587) | pCMV-Myc containing HCMV UL84 truncated sequence (400-587aa) | This study |
| pCMV-Myc-UL84(461-587) | pCMV-Myc containing HCMV UL84 truncated sequence (461-587aa) | This study |
| pCMV-Myc-UL84(Δ400-460) | pCMV-Myc containing HCMV UL84 truncated sequence (Δ400-460aa) | This study |
| pET28a-UL84 | pET28a containing HCMV UL84 full length sequence | This study |
| pCMV-Myc-UL44 | pCMV-Myc containing HCMV UL44 full length sequence | This study |
| pCDNA3.1-IE2 | pCMV-Myc containing HCMV IE2 full length sequence | This study |
| pET28a-UL44 | pET28a containing HCMV UL44 full length sequence | This study |
| pRK-11-Flag-FHL2 | pRK-11-Flag containing FHL2 cDNA full length sequence | This study |
| pRK-11-Flag-EZH2 | pRK-11-Flag containing EZH2 cDNA full length sequence | This study |
| pGEX-6p-1-FHL2 | pGEX-6p-1 containing FHL2 cDNA full length sequence | This study |
| pCDNA3.1-FHL2 | pCDNA3.1 containing FHL2 cDNA full length sequence | This study |
| pRK-11-Flag-FHL2(2-158) | pRK-11-Flag containing FHL2 truncated sequence (2-158aa) | This study |
| pRK-11-Flag-FHL2(2-216) | pRK-11-Flag containing FHL2 truncated sequence (2-216aa) | This study |
| pRK-11-Flag-FHL2(36-279) | pRK-11-Flag containing FHL2 truncated sequence (36-279aa) | This study |
| pRK-11-Flag-FHL2(95-279) | pRK-11-Flag containing FHL2 truncated sequence (95-279aa) | This study |
| pRK-11-Flag-FHL2(158-279) | pRK-11-Flag containing FHL2 truncated sequence (158-279aa) | This study |
| pRK-11-Flag-FHL2(ΔLIM2) | pRK-11-Flag containing FHL2 truncated sequence (Δ95-158aa) | This study |
| pRK-11-Flag-FHL2(Y97G) | pRK-11-Flag containing FHL2 a single point mutant Y97G | This study |
| pRK-11-Flag-FHL2(S98G) | pRK-11-Flag containing FHL2 a single point mutant S98G | This study |
| pRK-11-Flag-FHL2(S99G) | pRK-11-Flag containing FHL2 a single point mutant S99G | This study |
| pRK-11-Flag-FHL2(T112G) | pRK-11-Flag containing FHL2 a single point mutant T112G | This study |
| pCDNA3.1-TBP | pCDNA3.1 containing TBP cDNA full length sequence | This study |
| pGEX-6p-1-EZH2 | pGEX-6p-1 containing EZH2 cDNA full length sequence | This study |
| pCDNA3.1-IRF3 | pCDNA3.1 containing IRF3 cDNA full length sequence | This study |
| pCDNA3.1-c-Jun | pCDNA3.1 containing c-Jun cDNA full length sequence | This study |
| pCDNA3.1-p300 | pCDNA3.1 containing p300 cDNA full length sequence | This study |

This table lists all plasmid constructs used in this study, including basic expression vectors and recombinant plasmids containing full-length or truncated sequences of viral (UL84, UL44, IE2), host (FHL2, EZH2, IRF3, c-Jun, p300, TBP) genes and promoter reporter plasmid (NF-κB, IFN-beta). Tags such as Myc, Flag, His, and GST were used for expression and detection. Plasmids were generated based on standard molecular cloning methods, and all constructs were verified by DNA sequencing.
