# Supplementary material for: HCMV encoded UL84 hijacks FHL2 to suppress type I interferon production and enhance viral replication
Source: PLoS Pathog. 2026 Jan 26;22(1):e1013895. doi: 10.1371/journal.ppat.1013895 (PMC12863675; doi:10.1371/journal.ppat.1013895)
Supplement: Attachment — Submitted filename: Response to Reviewers (submit).docx [file ppat.1013895.s011.docx]

**Point-by-point response to reviewers’ comments**

**Part I – Summary**

Please use this section to discuss strengths/weaknesses of study, novelty/significance, general execution and scholarship.

**Reviewer #1:** Li et al provide a very nicely written, clear, interesting study of UL84 and FHL2 that is entirely suitable for publication in PLoS Pathogens. They show that FHL2 plays a novel architectural role in enhancing IFNb transcription upon infection with human cytomegalovirus or a number of other viruses, and provide detailed mechanistic analysis of how this occurs. Furthermore, they find that the HCMV UL84 protein interacts with FHL2 to help evade IFN signalling.

**Response: We sincerely appreciate the reviewer’s positive comments on our manuscript, as well as their recognition of the innovation and logical structure of our research.**

**Reviewer #2:** This manuscript by Ruilin et al. describes the role of HCMV-encoded UL84 as a negative modulator of the PRR-induced type I IFN response and identifies the FHL2 protein as a UL84 interacting protein, whose activity at the IFNbeta enhanceosome is hampered by UL84.

The interaction between UL84 and FHL2, identified in a Y2H assay, was confirmed with overexpression experiments via Co-IP in HEK293T and HFF cells, as well as confirmed by GST pulldown experiments, confirming a direct interaction. The authors further show that this interaction hampers assembly of the preinitiation complex at the Ifnb1 enhanceosome. They also show that FHL2 gets phosphorylated upon HCMV infection, which enhances Ifnb1 enhanceosome formation. While the authors present a lot of data characterising the interaction between UL84 and FHL2, mostly using overexpression studies, evidence showing the role of UL84-mediated FHL2 function during the IRF3-mediated type I IFN response in the HCMV infection context is missing. Most experiments are performed in presence of overexpressed (plasmid-derived) UL84, but reagents and methodologies do exist and are feasible to apply to show the relevance of this interaction in a more physiological context. in addition, several controls need to be included to show specificity for the observed phenotype.

**Response: We sincerely thank the reviewer for the constructive and insightful feedback.**

**（1）The reviewer mentioned that a lot of data was based on overexpression system, “evidence showing the role of UL84-mediated FHL2 function during the IRF3-mediated type I IFN response in the HCMV infection context is missing”.**

**To be objective, the HCMV Towne strain was utilized for experimental procedures. UL84 is an essential gene for HCMV Towne replication, as previously reported (PMID: 15367602); therefore, construction of UL84-deficient viral strains was not feasible. We also attempted to generate one and complement its function using UL84-expressing cells, but without success. Following the reviewers’ recommendations, we conducted supplementary experiments using siRNA targeting UL84 to validate its inhibition of HCMV-induced interferon production during viral infection, addressing the lack of defective virus and overexpression-only conditions (Figure 1D and 1E; Figure 2K and 2L). This approach has also been acknowledged in studies of other essential genes, as demonstrated in a publication in Cell Host & Microbe (2017, PMID: 28132838).**

**In the revised manuscript, now we have shown several data which was obtained under infection conditions, such as:**

a) **In Figure 1D and 1E, RT-qPCR analysis showed that siRNA knockdown of UL84 promoted HCMV induced transcription of IFNB1, ISG15, and IFIT1 genes in HFF;**
b) **In Fig 2D, the results of endogenous interaction assays show that UL84 and FHL2 exhibited significant enrichment in HFF cells infected with HCMV;**
c) **In Fig 2J-L, UL84 synonymous mutants (UL84-mut) can still significantly repress mRNA levels of IFN-β induced by HCMV infection in the presence of UL84-RNAi-#1. In contrast, UL84 (Δ400-460) mutants, which lack the interaction region with FHL2, fail to suppress IFN-β expression and promote efficient viral replication when co-expressed with UL84-RNAi-#1 in the HCMV infection context;**
d) **In Fig 3F, we assessed whether viral infection had any impact on the localization of FHL2;**
e) **In Fig 4G, knockout of FHL2 had no impact on the phosphorylation of IRF3, but the STAT1 phosphorylation was significantly inhibited in the context of HCMV infection;**
f) **In Fig 7C, UL84 specific siRNA-knockdown cell lines, by detecting changes in the phosphorylation of STAT1 and IRF3, we confirmed that UL84 may inhibit IFN-β transcriptional activation rather than the cascade signaling activation induced by viral infection;**
g) **In Fig 9A and B, we also detected the significant influence of UL84 and FHL2 interference via specific RNAi on IFN-β1 expression at the mRNA level after HCMV infection, as well as on viral titer.**

**（2）“In addition, several controls need to be included to show specificity for the observed phenotype”: As suggested by the reviewer, we performed several new experiments to add more controls, such as:**

a) **In the revised Figure 1B, we have added a time point of "0h" as a control.**
b) **In the revised Figure 5D, negative control has been added to confirm the co-immunoprecipitation result.**
c) **In the revised Figure 6D, negative control has been added to confirm the co-immunoprecipitation result.**
d) **In revised Figure 7A, we have added one more control group to show that overexpression of FHL2 significantly enhances the activity of the interferon promoter, while UL84 inhibits the activity of the interferon promoter.**
e) **In revised Figure 8A, a control group has been added to demonstrate that overexpression of FHL2 (T112G) significantly inhibits interferon promoter activity, whereas wild-type FHL2 enhances interferon promoter activity.**

**These data have been included in the revised manuscript.**

**Reviewer #3:** I read with interest the manuscript by Li et al. “HCMV encoded UL84 hijacks 1 FHL2 to suppress type I Interferon production and enhance viral replication”. In this article, the authors performed an impressive series of co-immunoprecipitations to identify novel functions for the host gene FHL2 and the HCMV gene UL84 in the regulation of IFNbeta expression. The most notable results are that (i) the authors show that FHL2 allows the transcriptional activation of IFNbeta by bridging the TATA binding protein with the IRF3-cJun-p300 complex at the IFNbeta promoter and (ii) that they show that UL84 disrupts the FHL2 binding to the abovementioned complex by interfering with FHL2 phosphorylation at the position T112. The authors show that UL84 overexpression enhances IFNbeta in cells infected with HCMV and that it affects viral replication. They also show that UL84 binding to FHL2 occurs at nucleotide positions 95-158 on the UL84 side and 400-460 on the FHL2 side. Finally, the authors state that the study provides a plethora of highly convincing biochemical data on the molecular interactions of FHL2 with host target proteins and DNA sequences in the IFNbeta promoter, as well as on the interactions with UL84. Hence, these data are well worth of publishing in general. The authors are less convincing in the virological interpretation of their findings. While UL84 is encoded and expressed by HCMV, the authors typically used experimental settings where cells were transfected with UL84 and then infected. Since almost all evidence in this article depended on the extrinsic addition of UL84 prior to virus infection, which is not the natural condition, it is unclear if UL84 affects IFNbeta expression in the context of virus infection. In fact, the only assay where a more natural setting was used is shown in panels 9A+9B and they show that the RNAi against FHL2 decreased the IFN responses and increased virus titers, although UL84 was present in this system. However, the manuscript title states that UL84 interaction with FHL2 enhances virus replication by decreasing IFNbeta. In light of my concerns, the effect of UL84-FHL2 interaction on virus replication seems insufficiently supported by hard evidence.

**Response: We sincerely thank the reviewers for their praise for our paper and their recognition of the research and logical structure of the research. We are very grateful for the constructive suggestions, which significantly improves the scientific accuracy and completeness of our research.**

**(1) The reviewer raised the concern that “it is unclear if UL84 affects IFNbeta expression in the context of virus infection”.**

**To be objective, the HCMV Towne strain was utilized for experimental procedures. UL84 is an essential gene for HCMV Towne replication, as previously reported (PMID: 15367602); therefore, construction of UL84-deficient viral strains was not feasible. We also attempted to generate one and complement its function using UL84-expressing cells, but without success. Following the reviewers’ recommendations, we conducted supplementary experiments using siRNA targeting UL84 to validate its inhibition of HCMV-induced interferon production during viral infection, addressing the lack of defective virus and overexpression-only conditions (Figure 1D and 1E). This approach has also been acknowledged in studies of other essential genes, as demonstrated in a publication in Cell Host & Microbe (2017, PMID: 28132838).**

**In the revised manuscript, now we have shown several data which was obtained under infection conditions, such as:**

a) **In Figure 1D and 1E, RT-qPCR analysis showed that siRNA knockdown of UL84 promoted HCMV induced transcription of IFNB1, ISG15, and IFIT1 genes in HFF;**
b) **In Fig 2D, the results of endogenous interaction assays show that UL84 and FHL2 exhibited significant enrichment in HFF cells infected with HCMV;**
c) **In Fig 2J-L, UL84 synonymous mutants (UL84-mut) can still significantly repress mRNA levels of IFN-β induced by HCMV infection in the presence of UL84-RNAi-#1. In contrast, UL84 (Δ400-460) mutants, which lack the interaction region with FHL2, fail to suppress IFN-β expression and promote efficient viral replication when co-expressed with UL84-RNAi-#1 in the HCMV infection context;**
d) **In Fig 3F, we assessed whether viral infection had any impact on the localization of FHL2;**
e) **In Fig 4G, knockout of FHL2 had no impact on the phosphorylation of IRF3, but the STAT1 phosphorylation was significantly inhibited in the context of HCMV infection;**
f) **In Fig 7C, UL84 specific siRNA-knockdown cell lines, by detecting changes in the phosphorylation of STAT1 and IRF3, we confirmed that UL84 may inhibit IFN-β transcriptional activation rather than the cascade signaling activation induced by viral infection;**
g) **In Fig 9A and B, we also detected the significant influence of UL84 and FHL2 interference via specific RNAi on IFN-β1 expression at the mRNA level after HCMV infection, as well as on viral titer.**

**(2) The reviewer has criticized “effect of UL84-FHL2 interaction on virus replication seems insufficiently supported by hard evidence”:**

a) **We constructed an expression plasmid containing UL84 mutations by introducing synonymous mutations into the region of the UL84-RNAi-#1 target sequence (UL84-mut), which preserved the UL84 amino acid sequence while conferring resistance to UL84-RNAi-#1 (Figure 2J). As shown in Figure 2K, UL84 synonymous mutants (UL84-mut) can still significantly repress mRNA levels of IFN-β induced by HCMV infection in the presence of UL84-RNAi-#1. In contrast, UL84 (Δ400-460) mutants, which lack the interaction region with FHL2, fail to suppress IFN-β expression when co-expressed with UL84-RNAi-#1. Meanwhile, viral titer assays confirmed that the C-terminal domain of UL84 (400-460 aa) was also required for efficient viral replication (Figure 2L). Collectively, these data imply the important role of the interaction between UL84 and FHL2 in subverting host immune defenses and supporting virus reproduction;**

**b) In Figure 7A, compared with wild-type, UL84 (Δ400-460) mutants, which lack the interaction region with FHL2, fail to suppress the dose-dependent enhancement of IFN-β promoter activity by FHL2;**

**c) In Figure 7E-G, UL84 but not its truncation mutant (Δ400-460) disrupts formation of the IRF3-c-Jun-p300 complex by interacting with FHL2;**

**d) Once FHL2^T112G^ lost the ability to interact with UL84, overexpression of UL84 did not further inhibit interferon levels, indicating that the regulation of UL84 on *IFN-B1* mRNA transcription acted through FHL2.**

**Therefore, we propose that UL84 likely affects viral replication through FHL2.**

**Part II – Major Issues: Key Experiments Required for Acceptance**

**Reviewer #1:** I have only relatively minor comments that need addressing prior to publication:

1. The authors say they ‘constructed a series of viral protein expression vectors and screened them using reporter assays for their abilities to regulate IFN-β promoter activity in HCMV-stimulated HFF cells’ – they should show this data

**Response: We thank the reviewer for this comment. We understand that the reviewer’ request is reasonable to some extent. We have identified a number of viral proteins that are involved in interferon inhibition, among which some research results are still in progress or have not been publicly published. Meanwhile, the present study focuses entirely on the investigation of interferon inhibition by UL84. We apologize for not being able to provide all the screening results and sincerely hope you can understand.**

2. I’m not sure I agree with the statement UL84 regulated IFN in a time-dependent manner; was there a significant difference in the IFNb-luc between time points (or the fold change between HCMV+vector vs HCMV+UL84) in Fig 1b?

**Response: We regret the inaccuracy in our original statement. The corrected version clarifies that HCMV infection leads to a time-dependent enhancement of IFN-β expression, and ectopic overexpression of UL84 in infected cells markedly inhibits the HCMV-induced interferon response. These revisions have been incorporated into the updated manuscript (lines 174~177).**

3. Initial experiments all use UL84 overexpression – one or two of these effects ought to be confirmed either using a UL84 deletion virus (if it is possible to generate one and complement it’s function using UL84-expressing cells) – OR using the efficient knockdown protocol they have in figure 7.

**Response: We sincerely thank the reviewer for this valuable and insightful comment. In this study, the HCMV Towne strain was utilized for experimental procedures. UL84 is an essential gene for HCMV replication, as previously reported (PMID: 15367602); therefore, construction of UL84-deficient viral strains was not feasible. We also attempted to generate one and complement its function using UL84-expressing cells, but without success. Following the reviewers’ recommendations, we conducted supplementary experiments using siRNA targeting UL84 to validate its inhibition of HCMV-induced interferon production during viral infection, addressing the lack of defective virus and overexpression-only conditions (Figure 1D and 1E; Figure 2K and 2L). This approach has also been acknowledged in studies of other essential genes, as demonstrated in a publication in Cell Host & Microbe (2017, PMID: 28132838).**

4. Authors should show the full data for the Y2H study, not just four ‘hits’. Also, where’s the confirmation of KPNA3 interaction with UL84 that is stated in Table S1. Should be shown.

**Response: We sincerely appreciate the reviewer’s suggestion. (1) In accordance with the reviewer’s comments, the full data of the yeast two-hybrid assay are presented in Figure S4A of the revised manuscript. (2) According to the suggestion, we now show the results confirming KPNA3 interaction with UL84 in Figure S4B of the revised manuscript.**

5. Why was EZH2 chosen as a control for the co-IP? Should say why.

**Response: We sincerely thank the reviewer for the question. To rule out nonspecific interactions, we considered it necessary to select a cellular protein as a negative control. Both FHL2 and EZH2 are transcriptional regulators involved in the initiation or repression of target gene transcription (PMIDs: 37148376, 10654935). Secondly, our previous study confirmed that EZH2 can interact with UL82 (PMID: 40818699), so this expression plasmid has been preserved in our laboratory. Furthermore, through preliminary interaction screening, we found no significant enrichment of interaction between EZH2 and UL84. Therefore, EZH2 was selected as a control in this study. In order not to cause the same confusion for other readers, we have provided clarifications in the revised manuscript (Line 217-220).**

6. Quality of the IF images in 2C was too poor to assess properly. Should show more cells, and potentially quantify the co-expression. In general the image quality was grainy – I appreciate this was only an initial submission, however high quality images must be provided for publication.

**Response: Thank you for your valuable comments. We have replaced and re-sized the picture to make it sharper.**

7. How is the effect on IFIT1 mediated? It is known to be directly responsive to IRF3 (i.e. prior to expression of IFN) – is this regulated similarly to IFNb? How about ISG15?

**Response: We thank the reviewer for raising this question. We examined IFIT1 and ISG15 in this study due to their status as interferon-inducible genes, with the aim of demonstrating that interferon activation and expression are indeed regulated by UL84.**

**According to the reports, Type I IFNs are secreted by virus-infected cells and subsequently bind the IFN-α/β receptor (IFNAR) on the cell membranes of the infected cells and neighboring cells. Activated IFNAR signals for JAK1 to phosphorylate STAT1 proteins, which form a complex with IRF9 called ISGF3 (PMID: 23203888). ISGF3 binds the ISRE within ISG promoters, increasing transcription and expression of hundreds of ISGs, including the typical ISG15.**

**As the reviewer mentioned, p-IRF3 also activates IFIT1, and we have not ruled out the possibility that UL84 regulates IRF3 signaling here. The detection of the total amount of p-IRF3, which remained unchanged in Figure 4F and 4G, as well as in Figure 7B-D, further confirms that the interaction between UL84 and FHL2 affects the transcription of interferon-β rather than the cascade signaling activation of viral infection. To prevent the same confusion for other readers, we have incorporated additional background information into the description of these results.**

8. “The results indicate that virus infection induces the phosphorylation and nuclear translocation of FHL2, which may serve as a cellular sensor-like role for detecting viral invasion” – or could it just be phosphorylated as part of a signalling cascade after sensing via known pathways? Similarly, the statement “function as a cellular sensor-like role in viral invasion” in line 427 and “we demonstrated that phosphorylation of FHL2 serves as a virus-triggered sensor-like role” (line 464) should be revised – they have not shown that FHL2 has a sensing role, more that it is involved in promoting IFNb transcription.

**Response: We sincerely appreciate the reviewer’s insightful comments and suggestions. Based on careful consideration of the results and the concept of sensors in pathogen biology, we agree with the reviewer’s suggestion that the role of FHL2 is merely to be phosphorylated and then translocated to the nucleus as part of a signaling cascade following sensing. These and similar statements (Line 40, 53, 55, 326, 352, 357, 477, 583) mentioned by the reviewer in the comments and elsewhere have been revised accordingly in the revised manuscript.**

9. The two statements “These results indicate FHL2 is a crucial mediator for virus-induced transcriptional activation of type I interferon” and “Considering FHL2 is proven to be a key regulator in the mechanism of IFN-β transcription” use results almost all determined earlier in the paper by FHL2 overexpression, apart from Figure 4D-E where the effect of si knock-down of FHL2 on STAT1 phosphorylation is weak. To confirm importance, they should knock it down our out in other experiments. They do a nice FHL2 knock-down for Figure 6B (or knock-out should be clarified?? I suspect this was a si knock-down should be stated as such. If knockout, provide methods), so should use this strategy in earlier sections of the paper to look at interferon regulation.

**Response: We sincerely thank the reviewer for these helpful suggestions.**

**(1) About “knock-out should be clarified?? I suspect this was a si knock-down should be stated as such. If knockout, provide methods”:**

**In Figure 6B, the FHL2 knock-out cell line was used for the ChIP assay. In accordance with your feedback, more experimental details have been provided in the revised Figure legends and Materials and Methods section.**

**(2) About “so should use this strategy in earlier sections of the paper to look at interferon regulation”:**

**According to the reviewer’s suggestion, we have newly conducted several supplementary experiments based on FHL2-KO cell line as in Figure 4C and Figure 4E of the revised manuscript. Just as the reviewers had predicted, after the knockout of FHL2, the levels of interferons and downstream ISGs induced by HCMV decreased more significantly compared to those after siRNA knockdown.**

**We greatly appreciate your constructive comments, which have greatly helped us improve the work.**

10. They should quantify all blots, at least for the supplementary, and state the number of replicates of every experiment in the figure legend.

**Response: We sincerely thank the reviewer for the suggestion.**

(1) **We have performed the necessary quantitative analysis on the Western blot data, and the corresponding values have been indicated to show the trend changes, such as in the revised Fig. 1 A; Fig 2J; Fig. 3 A-E; Fig. 4A-C, F, G; Fig. 7 A-C, F; Fig. 8A, D, F, G; Fig. 9A-C; Fig S5 A-D.**
(2) **Additionally, the missing clarifications regarding the number of replicates for some experiments have also been provided in the revised manuscript.**

**Reviewer #2:** Major points:

**1. a) Figure 1:** The luciferase reporter assays are conducted in HFF cells that were transfected with reporter plasmids plus empty vector or UL84, and then infected with HCMV with an MOI of 1, meaning that every cell must have gotten infected. Expression of UL84 upon transfection was quite strong and I am surprised to see that transfection has worked so efficiently. Can the authors please explain how they reached such high transfection efficiency? While I see that only cells can respond with an IFN response that received the reporter plasmids plus UL84, and not untransfected cells, this is not the case for HFF cells that were transfected, infected (MOI 1) and analysed with RT-PCR experiments for Ifnb1 (panel C). Such a strong phenotype can only be reached if most cells would have been transfected with UL84 - and I doubt that this can be achieved in HFF.

**b)** While UL84 also seems to inhibit the PRR-mediated IFN response upon IAV, EV71, HSV and ZIKV infection, it would be nice to include a control such as TNFalpha or other pro-inflammatory cytokines, which should not be affected (NF-kB reporter assay, RT-PCR in Fig S2 A-E). What could also be tested would be IFNAR signaling - this should not be affected by UL84 if it acts via FHL2 (IFNbeta stimulation, RT-PCR for IRF9 in Fig S2 F-J).

**c)** Panel A: Why can UL84 not be detected in HCMV infected cells (column 2)? According to the IP experiment in Figure 2 panel D, it should be expressed 24h post infection.

**Response:**

**a) In our prior experience, it was indeed observed that HFF cells, as primary fibroblasts, exhibited notably low transfection efficiency. Here, we have adopted the electroporation transfection method, which has been verified to be a relatively effective method despite its complex operation, according to previous reports (PMID: 39191758, 1116068). Therefore，plasmids transfection into HFF cells was in fact performed using the Bio-Rad Gene Pulser Xcell Electroporation System in this study. We have provided a fluorescence microscope image in Figure S3J to assess transfection efficiency, demonstrating that transfection of GFP-U84 using this method results in improved transfection efficiency compared to normal Liposome transfection. Although we described the methods in the original version of the figure legends, more relevant experimental details have been incorporated into the updated Figure legends and the Materials and Methods section.**

**b) During the early exploration, we indeed employed the NF-κB reporter assay and RT-qPCR to assess the expression levels of the NF-κB downstream genes IL-6 and TNF-α, thereby excluding the potential influence of UL84 on HCMV-induced inflammatory factors. These results have been included in the revised manuscript as Figure S2 B-U.** **In accordance with the reviewer’s recommendation, we have also conducted additional RT-qPCR experiments, as shown in Figure S2 G-U, which further indicate that UL84 cannot inhibit the host IFN-α-mediated antiviral response against different viruses.**

**c) In fact, here we used anti-Myc antibodies to detect the gradient-transfected Myc-tagged UL84. Therefore, the endogenous expression of UL84 by HCMV was not detectable. We sincerely apologize for the inaccurate labeling, which may have caused confusion for readers. The UL84 label has been revised to Myc-UL84, and the relevant experimental details have been incorporated into the updated figure legend.**

2.The IRF3-mediated IFN response is induced shortly after infection. I find no proof in the manuscript that UL84 is expressed at this time point. A kinetic experiment should be shown analysing UL84 expression at different time points post transfection, together with p-IRF3 IBs.

**Response: We sincerely thank the reviewer for this valuable and insightful comment. Based on this suggestion, we have conducted the supplementary experiments as Figure S1A and 1B, the kinetics of UL84 expression at different time points post transfection were detected by RT-qPCR and Western blot. Results showed that low levels of UL84 protein expression were detected as early as 3h in HCMV-infected HFF cells, as reported in a previous article (PMID: 1309892). Meanwhile, the p-IRF3 level was immediately upregulated upon virus infection and reached a plateau at 9 hpi.**

3. Figure 2: The IF shown in panel C should be conducted with endogenous FHL2 instead of overexpressed FHL2.

**Response: According to the reviewer’s suggestion, we have replaced the original Figure 2C in the revised manuscript to show the results obtained with endogenous FHL2. Similar to the situation observed upon their overexpression, UL84 and FHL2 were found to mainly co-localize in the cytoplasm, with a small amount of FHL2 imported into the nucleus to co-localize with UL84.**

**It should be noted that, to make the article’s narrative more fluent and to avoid redundancy in the figure, we did not show the colocalization results of fully endogenously expressed UL84 and FHL2 under viral infection here; instead, these results are presented in Figure 3F.**

4. If UL84 indeed inhibits the IFN response, cells transfected with UL84 should show lower titers of IAV. This would be an experiment that would convincingly show that it has a biological effect.

**Response: We sincerely thank the reviewer for the suggestion. In response, we have now performed additional experiments to investigate the effect in cells transfected with UL84 on the titer of IAV. In Figure S2U, the data indicate that cells overexpressing UL84 indeed show higher titers of IAV. Our results suggest that UL84 generally inhibits the host IFN-β-mediated antiviral response against different viruses.**

5. Figure 4 A, lane 2: why is endogenous FHL2 expression not induced upon HCMV infection (24h p.i.)? I would suggest to do this assay at the time point when it was shown to be induced, 4-6 hpi (Figure 3). As shown in panel B of this figure, the IFN response is highest 6 h pi and declines afterwards. For panel B, it would be important to include an IB control showing endogenous UL84 expression and include an FHL2 and GAPDH IB.

**Response: (1) We are extremely grateful to the reviewer for pointing out the mistake we overlooked. It might have been caused by a problem with sample loading or exposure of ECL of lane 2 during the experiment. We have repeated the experiments and provided updated data including Western blot results. FHL2 indeed enhance the activation of IFN-β promoter induced by HCMV in a dose-dependent manner, while the expression of FHL2 was increased during HCMV infection. (2) As suggested by the reviewer, the viral infection stimulation time in this study is indeed 6 hours, at which point the best stimulation effect was observed, as shown in Fig. 1B, where we performed a time-course experiment. We also reviewed the experimental records of the relevant students to confirm this; therefore, the mention of 24 hours here was a writing error, which has been corrected in the revised manuscript.**

**(3) As suggested by the reviewer, the IB controls showing endogenous UL84 expression and including an FHL2 and GAPDH IBs have been added in the revised Figure 4B and 4C.**

6. Figure 4 D+E: siRNA transfection targeting FHL2 has no effect on p-STAT levels. This is not mentioned in the result section. The same is true for overexpression of FHL2 - no effect on p-STAT levels. However, here the authors claim that p-STAT levels are affected, with which I disagree with. While P-IRF3 levels should not be affected, p-STAT levels should be affected of FHL2 is important for type I IFN induction. This experiment weakens the study significantly and it is irritating that the data is interpreted entirely differently by the authors.

**Response: We gratefully acknowledge the reviewer for raising this question. Indeed, our prior analysis of repeated experimental trials demonstrated that both siRNA knockdown and overexpression of FHL2 exerted a significant effect on p-STAT levels. It may not seem so obvious in the picture, so we performed a quantitative analysis of p-STAT protein levels using Image-J software. Meanwhile，other reviewers suggested that it would be better to conduct this experiment using knockout cells rather than siRNA, so we repeated the experiment as requested. We observed that both FHL2 knockout and overexpression exert a stronger effect on pSTAT1 levels, which further confirms our conclusions. We have updated Figure 4 F-G with the new results, and revised the corresponding descriptions in the manuscript.**

7. Figure 5 C: is not labeled comprehensively. Lacks an EV control (no FHL2 control).

**Response: In Figure 5D of the revised manuscript, in accordance with the reviewer’s suggestion, we have repeated the experiments and added the Empty Vector control group.**

8. ChIP assay: I do not think one can conclude that FHL2 binds to the region -84--+46 based on this experiment. This figure would benefit from a comprehensive scheme of the Ifnb1 promoter/enhancer, the IRF3 etc. binding sites and the primer binding sites.

**Response: We thank the reviewer for the suggestion to improve the readability of the manuscript. According to the information from the** **website （Login to https://epd.expasy.org/epd/ and retrieve the Homo sapiens IFNB1 promoter sequence (-499 to +100)）and based on conserved sequence 5'-AANNGAAA-3' recognized by IRF3 (PMID: 9422515), conserved sequence 5'-TGACATAG-3' recognized by c-Jun (PMID: 10848607), and conserved sequence 5'-GGGRNNYCC-3' recognized by NF-κB (PMID: 11533489), we have created a schematic diagram as Figure 5A of the revised manuscript to help readers understand our experimental design.**

9. Panel A should be labeld more clearly (are all lanes HCMV-infected?). The control FLH2 + EV is missing (as control for FHL2 + UL84).

**Response: We sincerely apologize for the lack of clarity in the original labeling, which may have led to confusion among readers. In response to the reviewer’s feedback, we have revised the labeling, repeated the experiment, and incorporated an additional control group (FLH2 + EV) to strengthen the experimental design. These results further suggest that UL84 inhibits interferon transcription through the interaction with FHL2.**

10. Figure 7: Since the UL84 siRNA works well, this experiment should be conducted with UL84 expressed in the virus context. Without UL84 plasmid-derived overexpression. As control, pIRF3 (should not change) and pSTAT (should be affected) IBs should be done.

**Response: We sincerely thank the reviewer for this important suggestion. In accordance with the reviewer’s comments, we have repeated the experiments and examined the change in p-IRF3 levels as a control. The results demonstrated that there was no significant change in the total amount of phosphorylated IRF3 at 6 hours post-infection, thus further supporting the conclusion that UL84 regulates interferon beta expression at the transcriptional level. Meanwhile, according to the recommendations of other reviewers, UL84 siRNA and plasmid-derived overexpression under the condition of viral infection are presented as two separate figures (Figure 7B-D) in the revised manuscript.**

11. The authors use HCMV strain TOWN throughout their study. Since UL84 is esssential for viral replication, a knockout virus could not be tested and experiments were conducted with overexpression of UL84 or siRNA targeting UL84. However, HCMV strain TB40 can replicate without UL84 and hence it would be possible to test whether HCMV lacking UL84 induces a stronger IFN response compared to WT virus.

**Response: We sincerely thank the reviewer for the good suggestion. Unfortunately, we do not currently have a collection of EL350 strains containing the TB40 BAC in our laboratory, and we have tried but have been unable to obtain them from other sources in the near future; therefore, we cannot construct TB40 UL84 mutants at present. We have also read the articles reporting that UL84 is not essential for TB40, but it can still affect the viral replication efficiency to some extent. This observation strongly supports the viewpoint of this study that UL84 has functions beyond its traditional role as a protein considered essential for replication. We gratefully acknowledge the reviewer for providing this valuable information. The two related articles about TB40 UL84 will be incorporated into our references and discussed in the Discussion section to serve as validation and support for subsequent work (Line 681,705).**

12. Figure 8 panel A lacks the control HCMV + EV.

**Response: In Figure 8A of the revised manuscript, in accordance with the reviewer’s suggestion, we have repeated the experiments and added the HCMV + EV control group.**

13. Figure 9 lacks IB controls which would be essential to validate the presented results.

**Response: We sincerely thank the reviewer for the suggestion. We have repeated the experiments and added the corresponding Western blot data for the controls (Figure 9A–C) in the updated figures.**

14. Manuscript lacks information about the number of biological and technical replicates.

**Response: We truly appreciate the reviewer for the careful review of our manuscript, which will greatly help improve the quality of our work. The missing clarifications regarding the number of replicates for some experiments have now been clearly indicated in the Figure legends and Materials and Methods section of the revised manuscript.**

**Reviewer #3:**

**1.** Show effects of UL84 in the context of virus infection. A virus mutant with a full deletion of UL84 may is not easy to generate due to its essential requirement for viral DNA replication, but it may be grown o na complementing cell line. It may also be possible to generate a mutant virus where UL84 can still act with UL44 on virus DNA replication but is unable to bind to FHL2. The authors show in figure 2 that a deletion of nucleotide positions 95-158 abrogates its binding to FHL2. Hence, a mutant virus lacking only these nucleotides may be viable, which would allow the identification of UL84 effects on the FHL2-IFNbeta signaling axis.

**Response: We sincerely thank the reviewer for this valuable and insightful comment. In this study, the HCMV strain was utilized for experimental procedures. UL84 is an essential gene for HCMV replication, as previously reported (PMID: 15367602); therefore, construction of UL84-deficient viral strains was not feasible. We also attempted to generate one and complement its function using UL84-expressing cells, but without success.**

**During the period of revising the manuscript, we also took the opportunity to attempt to construct a mutant virus with a deletion of amino acids at positions 400-460 to abrogate its binding to FHL2. Unfortunately, no progeny virus could be generated either. The possible reason is that the deletion of this sequence might disrupt the correct folding of UL84 or affect its other functions. According to the reports, the partially overlapping sequences on both sides of this region may be involved in other functions. For instance, aa 114–135 contains a leucine zipper motif related to the binding of IE2 (PMID:15308715); aa 101-200 might contain its own binding region (PMID:15308715). Meanwhile, in Figure 9B, we investigated whether the interaction between UL84 and FHL2 directly affected viral DNA replication using an *in vitro* transient transfection-based replication reporter assay. The results showed that the T112 residue mutation in FHL2, which abolished its interaction with UL84, enhanced viral DNA replication, thereby inversely demonstrating that the interaction between UL84 and FHL2 may directly participate in regulating viral replication. This is consistent with previous reports that UL84 is an essential replication protein.**

**According to the recommendation of other reviewers, we conducted supplementary experiments using siRNA targeting UL84 to validate its inhibition of HCMV-induced interferon production during viral infection, addressing the lack of defective virus and overexpression-only conditions (Figure 1D and 1E; Figure 2K and 2L). This approach has also been acknowledged in studies of other essential genes, as demonstrated in a publication in Cell Host & Microbe (2017, PMID: 28132838).**

2. It is also possible that UL84 expression during the lytic virus cycle occurs too late to affect IFNbeta responses. This would invalidate a major claim from the title of the manuscript. Hence, it may be interesting to explore the effects of UL84 in models of HCMV latency and reactivation, where UL84, if expressed early during reactivation, may affect IFNbeta responses and facilitate the full blown virus reactivation. At a minimum, these items need to be discussed as alternative scenarios, while the title is changed to reflect more accurately the available evidence provided in the manuscript.

**Response: We sincerely thank the reviewer for this thoughtful comment. In fact, UL84 is not a gene expressed very late. According to reports, changes in viral mRNA levels can be detected as early as 2.5 hours post-infection in HCMV-infected host cells (PMID: 1309892). Our experiments also demonstrated a protein signal at 3 hours post-infection (Figure S1B). We have indeed recently reported some research on the HCMV latency and reactivation (PMID: 40818699, 40521898), but whether UL84 is related to the latency and reactivation of the virus by regulating the interferon response still needs further exploration.**

**Part III – Minor Issues: Editorial and Data Presentation Modifications**

**Reviewer #1:** Minor comments

**1.** Line 270 ‘enhancersome’ spelled incorrectly.

**Response: We thank the reviewer for pointing out** **this mistake. To address this, we have carefully reviewed the entire manuscript to ensure there are no spelling errors (lines 350).**

**2.** ‘did not be co-immunoprecipitated with TBP’ -> ‘was not co-immunoprecipitated…’

**We sincerely appreciate the reviewer for pointing out the grammatically incorrect sentences, which greatly improved our manuscript. We have revised the sentences (lines 400) and carefully checked the language issues throughout the manuscript again.**

**Reviewer #2:** Other comments:

**1.** The information provided in the M+M section is not sufficient to recapitulate the experiments in most sections:

a) The origin of the cell lines is not provided (HFF and 293-T). Ideally, also the passage number should be provided.

b) Details on the transfection of HFF cells are not provided. HFF are difficult to transfect and I do not know any lab that has schieved it to a level as high as shown here. It would be great to share the protocol with the scientific community. How many microgramms of DNA were transfected per well? This should also be stated for the titration experiments.

c) Information on the luciferase plasmid is not sufficient. Which sequence of the IFNb1 promoter/enhancer is inserted upstream of the luciferase gene?

d) Information on the UL84 plasmid is not sufficient. Is it UL84 derived from TOWN?

e) The reference ID of the TOWN BAC needs to be provided. Was the TOWN-BAC verified by sequencing?

f) Some IPs and subsequent immunoblots were performed with the same antibody and are impressively clean. It would be good to state in the M+M section which antibody was used for which application and also mention the species it was made in (IP, IB).

**Response: We sincerely appreciate the reviewer for the careful review of our manuscript, which greatly improves the rigor of our work. In accordance with the recommendations, we have revised the relevant content in the Materials and Methods and Figure legends section to enhance clarity and consistency as follows:**

a) **Human foreskin fibroblasts (HFF cells, passages 9 to 14) and 293T were obtained from the American Type Culture Collection (ATCC) (Manassas, VA, USA).**
b) **HFF cells are indeed a type of cell that is widely acknowledged as being difficult to transfect in this field. Here, we have adopted the electroporation transfection method, which has been verified to be a relatively effective method despite its complex operation, according to previous reports (PMID: 39191758, 1116068). Therefore，plasmids transfection into HFF cells was in fact performed using the Bio-Rad Gene Pulser Xcell Electroporation System in this study. In the revised manuscript, we presented a distinct description of the transfection method for HFF cells and incorporated additional details.**
c) **The sources and construction information for the IFN-β-luciferase reporter, NF-κB-luciferase reporter, and other plasmids have been updated in the revised manuscript (lines 745-752).**
d) **The UL84 gene was amplified from HCMV (Towne BAC), which were kindly provided by Prof. Fenyong Liu (University of California, Berkeley, CA, USA). Subsequently, the UL84 gene was cloned into the pCMV-Myc plasmid vector ( Addgene, Catalog Number: 631604).**
e) **HCMV (Towne BAC) (GenBank: AC146851.1) were kindly provided by Prof. Fenyong Liu (University of California, Berkeley, CA, USA). The Towne BAC was sequenced by Tsingke Biotechnology company to validate its sequence information and accuracy.**
f) **The company, Cat. No., and properties of antibodies for all relevant immunoprecipitation and immunoblotting experiments were indicated.**

**2.** I was intrigued to see the molecular weigth difference of unphosphorylated FHL2 (32 kDa) and its phosphorylated version which runs as high as 95 kDa (Figure 3 panel G-K). Can the authors please explain this phenomenon?

**Response: Due to the unavailability of a commercially specific antibody for detecting FHL2 protein phosphorylation, we employed Phos-tag SDS-PAGE to detect potential phosphorylation of FHL2. Phos-tag SDS-PAGE is a phosphor-affinity electrophoresis technique that enables the separation of phosphorylated and non-phosphorylated proteins using standard SDS-PAGE procedures. This technique employs a separation gel in which acrylamide is copolymerized with Phos-tag Acrylamide (a phosphate-capturing molecule). Due to the reversible binding between phosphorylated proteins and the immobilized Phos-tag in the gel during electrophoresis, phosphorylated proteins migrate more slowly than their non-phosphorylated counterparts. Accordingly, the final detected molecular weight of FHL2 exhibited a significant upward shift. Our Western blot results indicate the presence of at least 3 phosphorylation sites in FHL2.**

**3.** Did the authors do an alphafold prediction with UL84 and FHL2 to analyse their interaction?

**Response: Because in previous studies, we conducted truncation interaction analyses using Co-IP experiments based on reported domains and identified the interaction regions between UL84 and FHL2, we did not perform AlphaFold predictions for the UL84–FHL2 complex. Following the reviewer’s suggestion, in the updated manuscript, we have added AlphaFold-based predictions for UL84 and FHL2. As shown in the Interaction Diagram (Figure 2I) and the Molecular Docking Result (Figure S6A), the simulation results are consistent with the previously identified truncated interaction regions. A description of molecular docking simulation between UL84 and FHL2 has been added to the revised manuscript.**

**4.** The depth of information about the Y2H assay are scarce and the Y2H assay results are currently only listed in a supplementary table. More detail (in the method section as well as the result section) should be shown. Ideally, results of the Y2H assay should be shown as figure 1 or 2.

**Response: Thanks for the valuable comments. According to the reviewer’s comments, the results of the Y2H experiments between UL84 and FHL2 are shown in Figure S4A. We also show the results confirming KPNA3 interaction with UL84 in Figure S4B of the revised manuscript. Meanwhile, we have incorporated more experimental details and result descriptions in the Methods and Results sections.**

**The high-throughput Y2H screening was our very early work, and we merely regarded the screening results as the basis for subsequent research. In recent years, based on these screenings, we have published many research papers (PMID: 34747321, 33967989, 29377960). Secondly, after the Y2H screening, it is usually still necessary to conduct verification using Co-IP and GST/His pull-down assays in human cells and under infection conditions. Therefore, we considered including the relevant results as supplementary figures.**

**5.** In their introduction, the authors could elaborate more about UL84.

**Response: We thank the reviewer for this helpful suggestion. In response to these comments, we have revised the introduction section by integrating and summarizing information on the structure and biological functions of UL84, while also updating the reference list accordingly.**

**Minor comments (language related):**

1. abstract: "...binds to the cellular receptor..." - to what are the authors refering to here?

**Response: The confusing sentence has been rephrased according to the reviewer’s suggestion.**

2. Line 66 and onwards: The authors cite >20 year old papers regarding the IFNbeta enhanceosome. While it is important to cite original papers and give credit to those authors, a more recent review article on IRF3 could be cited as well (for example Schwanke et al 2020 doi: 10.3390/v12070733).

**Response: We sincerely thank the reviewer for this helpful suggestion. In the revised manuscript, we have cited more recent references, including the recommended ones.**

3. Line 60: ..."upon the viral binding of PRRs..." is phrased in a strange manner - the virus itself does not bind to PRRs, rather viral ligands or PAMPs.

**Response: The sentence has been revised according to the reviewer’s suggestion.**

4. Line 97: please write out for what LIM stands for

**Response: We sincerely thank the reviewer for the suggestion. The revised version of the manuscript now includes the full name of the first-mentioned proper noun LIM, along with relevant background information (Lines 101-104).**

5. Line 118: " HCMV is the largest herpesvirus in the genome..." needs to be rephrased.

**Response: Human cytomegalovirus (HCMV) has the largest genome in the Herpesviridae family. The sentence pointed out by the reviewer has been revised.**

6. Line 582: typo (Reagents)

**Response: All the typos in the entire manuscript have been corrected.**

7. Line 665: strange phrasing ("there are six main concerns...")

**Response: We sincerely appreciate the reviewer’s efforts in pointing out the confusing sentences in our language expression. We have carefully revised similar erroneous phrasing to enhance clarity and accuracy.**

8. Line 703-704: strange sentence

**Response: We sincerely appreciate the reviewer for the careful review of our manuscript, which greatly improves the quality of our work. These sentences mentioned by the reviewer, as well as the entire manuscript has been thoroughly revised to improve language accuracy and clarity.**

9. Figure 2 panel E and G should be labeled more clearly. Panel D: MW marker is missing.

**Response: We have enlarged the font size of the labels and uploaded clearer images. The missing MW marker has also been added.**

10. Line 270: enhanceosome

**Response: We thank the reviewer for pointing out** **this mistake. To address this, we have carefully reviewed the entire manuscript to ensure there are no spelling errors.**

**Reviewer #3:**

1. There is very little information provided about the plasmids pcDNA3.1-UL84 and pGL3-IFN-beta. Wat promoter is used in the former one? Is the latter one encoding the IFNbeta promoter? Which part of the promoter? Does it also express IFNbeta or just the luciferase? What is the evidence that it matches the patter of IFNbeta gene expression at the native site? If there is a previous publication about these plasmids, it needs to be more clearly cited. If they are new constructs, more details on their characterization need to be provided.

**Response: We sincerely apologize for the omission of the sources and construction details of these key materials in the initial manuscript. Sequencing of the pGL3-luci-IFN-beta plasmid confirmed that it contains two interferon-stimulated response element (IRSE) repeats, with each core element harboring conserved sequences recognized by c-Jun, IRF3, and p65, which is consistent with the core element sequence of the IFN-β promoter (NM_002176.4). All relevant information has now been thoroughly incorporated into the revised version.**

2. pGL3-IFN-beta is mentioned in some part of the text, but not in M&M, where the only plasmid with a similar name is the pGL3-luci-IFN-beta. Please adjust and standardize this nomenclature.

**Response: We sincerely thank the reviewer for carefully pointing out the issues regarding plasmid information. These reporter gene plasmids were previously obtained as gifts from academic collaborators and have been maintained in our laboratory since their acquisition. To address this, we have thoroughly reviewed the entire manuscript to ensure that all plasmid information has been updated in the Materials and Methods section.**

3. What MOI was used in figures 1D-1G?

**Response: The MOI values used were as follows: IAV (MOI = 0.5), EV71 (MOI = 1), HSV (MOI = 1), and ZIKV (MOI = 1). These parameters clearly indicated in the figure legends for Figures 1F–1I.**

4. 293T cells (HEK 293 T cells?) are used for cotransfections of UL84 and FHL2. It would be useful to state once they are introduced for the first time if these cells express FHL2 at low levels or not at all.

**Response: (1) The 293T cells commonly referred to in routine experiments are in fact HEK 293T cells, which we have corrected to the standard designation. (2) The natural abundance of FHL2 was measured in other virus-infected cells, where it was relatively low, and virus infection can stimulate the expression of FHL2 (Figure 3A–3E). The interaction between UL84 and FHL2 in endogenous conditions was verified in HFF cells. Here, HEK 293T cells were mainly used for *in vitro* Co-IP validation of UL84 and FHL2, and therefore had to be ectopically transfected with full-length or truncated FHL2-expressing plasmids.**

5. Please indicate molecular weights in figure 2D

**Response: We have checked all the bands in the Western Blots, including those in Figure 2D, to ensure that they are all indicated with their molecular weights.**

6. Lines 301 state that indicate that “all four predicted sites (Y97, S98, S99, and T112) within the LIM2 domain are essential for FHL2’s interaction with IRF3, c-Jun, or p300 during the assembly of the IFN-β enhanceosome complex.” However, it may also be possible that some or all of these sites are important for correct FHL2 protein folding, rather than a direct interaction with the target proteins.

**Response: We thank the reviewer for this thoughtful comment. We are currently unable to definitively determine whether the individual mutations at these four sites impact the proper folding of FHL2. However, as demonstrated in Figure 6F, these point mutations do not impair its interaction with TBP. Based on this evidence, it can be reasonably inferred that these mutations are unlikely to cause misfolding of FHL2. To address this question, we performed stability analysis following molecular dynamics simulations and calculated the following indicators: radius of gyration (Rg), root mean square deviation (RMSD), root mean square fluctuation (RMSF), solvent-accessible surface area (SASA), free energy landscape (FEL) and hydrogen bond (Hbond) analysis. Radius of gyration (Rg) analysis showed that wild-type and mutant proteins generally showed a gradual shrinkage trend, and the overall structure was compact and stable (Figure 5H). The root means square deviation (RMSD) results showed that the overall protein structure tends to be stable: the FHL2 wild-type structure is the most convergent and stable, while the FHL2^T112G^ mutant structure fluctuates the most and is the least stable (Figure 5I). The root means square fluctuation (RMSF) values fluctuated around 0.75 nm, suggesting minimal changes in the secondary structure conformation (Figure 5J). The solvent-accessible surface area (SASA) maintained a level of ~130 nm²(±10%) after 40 ns, with a fluctuation amplitude < 10 nm²and no significant variation throughout the 200 ns simulation (standard deviation<1%). The hydrophobic surface area accounted for ~50%, the hydrophilic surface area for ~50%, and the total surface area for ~100%, indicating a balanced interface between the wild-type and mutant proteins (Figure 5K). This result has been added to the manuscript.**

7. Figures S4 and S5 seem to be swapped in the text.

**Response: Thanks to the reviewer for pointing out this mistake. These two figures were indeed misplaced, and we have now corrected their order.**

8. In figure 6B, there is still a band for TBP, though it is less pronounced. A similar reduction in signal is observed for Jun and p300. So, the text in lines 318-320 (“when we knocked out the endogenous FHL2, IRF3 failed to be co-immunoprecipitated with TBP, although it still recruited p300 and c-Jun to the IFN-β promoter”) does not accurately describe the figure 6B.

**Response: We gratefully acknowledge the reviewer’s careful observations and have accordingly revised the description of this result to enhance its accuracy (Line 437-439).**

9. The text in lines 320-21 “Molecular docking of complex form the IRF3-FHL2-TBP demonstrates that IRF3 specifically recognizes the N-terminal LIM domain of FHL2,” is confusing, please rephrase.

**Response: We sincerely appreciate the reviewer’s efforts in pointing out the confusing sentences in our language expression. We have carefully revised similar erroneous phrasing to enhance clarity and accuracy (Line 439-441).**

10. In figure 7H, it appears that an IP was performed on UL84, but the text states that it was done on FHL2. If the IP was on FHL2, how comes the band for FHL is absent in the WB in the second row, second column?

**Response: We sincerely apologize for the incorrect labeling of the image here. The IP was indeed performed on UL84, and the error in the label has been corrected in the updated Figure 7H.**

11. How comes HCMV infection induces IFNbeta in the second column of figure 8A, even though HCMV encodes the UL84 gene?

**Response: We thank the reviewer for the question. The interferon response triggered by viral infection may be initiated at multiple stages of the viral life cycle. For instance, as previously reported, it can be activated during the stage of cell adsorption through NF-κB signaling (PMID:7636984), or upon entry of the viral genome into the cytoplasm when it is detected by cellular DNA sensors (PMID:16545883). In this study, UL84 and FHL2 mainly regulates the transcription of IFN-β. Furthermore, as shown in Figure S1B, UL84 expression can only be detected by Western blot at 3 hpi, followed by gradual accumulation. Notably, its expression level does not immediately reach that observed with plasmid-mediated overexpression. Therefore, in the case of natural infection, although the virus expresses UL84 in the early stage, it is not sufficient to completely suppress the production of IFN-β.**

12. While the PKC inhibitor LY33531 in figure 8F shows an effect on nuclear translocation of FHL2, this experiment would be corroborated had the authors used a PKC-specific siRNA.

**Response: In accordance with the reviewer’s comment, we have performed supplementary experiments, and the results are presented as in Figure 8G and 8H. It can be seen that the treatment with PKC-specific siRNA and inhibitor has a similar effect.**

13. The data in figure 9B should be shown as growth curves on a logarithmic scale, not using a linear scale. Virus growth is exponential so should be the scale that display it.

**Response: Thank the reviewer for pointing out this issue. The amplification of the virus proceeds in powers of 2. Consequently, the vertical axis of the virus growth curve ought to be presented in logarithmic form. We have reprocessed the original data and now present the changes in viral proliferation in the form of a logarithmic coordinate axis.**

14. The figure 9C is confusing – they say that they measure OryLit replicative activity by using a luciferase reporter, which is indicative of transcription and gene expression, not of DNA replication. Please explain this better. This growth curve should be performed more ideally over a period of a week, not only for 4 days, because HCMV replicates slowly in cell culture.

**Response: Here an *in vitro* transient transfection-replication reporter assay (PMID: 33967989, 39191758) was adopted to investigated whether the interaction between UL84 and FHL2 directly affected viral DNA replication, since it was reported that UL84 is required oriLyt-dependent DNA replication. Briefly, the HCMV replication origin DNA (OriLyt) was inserted into the pGL3-promoter vector (Addgene: 48743) at the multiple cloning site (MCS) upstream of the SV40 promoter, as previously described for the construct designated pGL3-14 (PMID: 33967989). The vector enables constitutive firefly luciferase expression. The copy number of oriLyt replication is positively correlated with luciferase expression. Then, HFF cells were co-transfected with plasmids containing the HCMV oriLyt (pGL3-14) and plasmids expressing six core replication proteins and three auxiliary proteins. The effect of UL84 and FHL2 on oriLyt-dependent replication after co-transfection can be measured by dual-luciferase reporter assays. Therefore, the replication process of HCMV is simulated using an *in vitro* multi-plasmid system, which differs from the actual viral infection process.**

**To prevent other readers from experiencing similar confusion, we have provided additional descriptions of the experimental methods and objectives in the Results section, Figure legends, and Materials and Methods section.**

15. Numerous mistakes in sentence structure were observed in lines 99, 103, 106, 109, 118 119-120, 317, 320, 359, 380, 381, …

**Response: We sincerely appreciate the reviewer for the careful review of our manuscript, which greatly improves the quality of our work. These sentences mentioned by the reviewer, as well as the entire manuscript has been thoroughly revised to improve language accuracy and clarity.**
